# Virological Evaluation of Avian Influenza Virus Persistence in Natural and Anthropic Ecosystems of Western Siberia (Novosibirsk Region, Summer 2012)

**DOI:** 10.1371/journal.pone.0100859

**Published:** 2014-06-27

**Authors:** Maria A. De Marco, Mauro Delogu, Mariya Sivay, Kirill Sharshov, Alexander Yurlov, Claudia Cotti, Alexander Shestopalov

**Affiliations:** 1 Laboratorio di Genetica, Istituto Superiore per la Protezione e la Ricerca Ambientale (ISPRA), Ozzano Emilia (BO), Italy; 2 Department of Veterinary Medical Sciences, University of Bologna, Ozzano Emilia (BO), Italy; 3 Research Center of Clinical and Experimental Medicine, Siberian Division of the Russian Academy of Medical Sciences, Novosibirsk, Russia; 4 Institute of Systematics and Ecology of Animals of the Siberian Branch of the Russian Academy of Sciences (RAS), Novosibirsk, Russia; 5 Research Division, Novosibirsk State University, Novosibirsk, Russia; Duke-NUS Graduate Medical School, Singapore

## Abstract

**Background:**

Wild aquatic birds, reservoir of low-pathogenicity (LP) avian influenza viruses (AIVs), congregate in huge numbers in Western Siberia wetlands, where major intra- and inter-continental bird flyways overlap. In 2005 and 2006, highly pathogenic (HP) AIV H5N1 epizootics affected wild and domestic birds in the Novosibirsk Region. In 2012, we evaluated AIV persistence in Siberian natural and anthropic ecosystems.

**Methodology/Principal Findings:**

In Novosibirsk Region, 166 wild birds ecologically linked to aquatic environments and 152 domestic waterfowl were examined for AIV isolation in embryonating chicken eggs. Biological samples were obtained by integrating the conventional cloacal swab collection with the harvesting of samples from birds' plumage. Haemagglutinating allantoic fluids were further characterized by serological and molecular methods. In August-September 2012, 17 AIVs, including three H3N8, eight H4N6, two H4N?, one H2N?, one H?N2, and two unsubtyped LPAIVs, were isolated from 15 wild ducks. Whereas comparable proportions of wild *Anseriformes* (n.118) tested virus isolation (VI)-positive from cloaca and feathers (5.9% *vs* 8.5%) were detected, the overall prevalence of virus isolation, obtained from both sampling methods, was 2.4 times higher than that calculated on results from cloacal swab examination only (14.4% *vs* 5.9%). Unlike previously described in this area, the H4N6 antigenic subtype was found to be the prevalent one in 2012. Both cloacal and feather samples collected from domestic waterfowl tested VI-negative.

**Conclusion/Significance:**

We found lack of evidence for the H5N1 HPAIV circulation, explainable by the poor environmental fitness of HPAIVs in natural ecosystems. Our LPAIV isolation data emphasise the importance of Siberia wetlands in influenza A virus ecology, providing evidence of changes in circulation dynamics of HN antigenic subtypes harboured in wild bird reservoirs. Further studies of isolates, based on bioinformatic approaches to virus molecular evolution and phylogenesis, will be needed to better elucidate mechanisms involved in AIV perpetuation in this area.

## Introduction

Western Siberia wetlands of the Novosibirsk Region are an important sanctuary for wild aquatic birds representing major reservoirs of influenza A virus gene pool, from which novel influenza viruses can emerge to infect other avian and mammalian species, human beings included [1]. The natural virus perpetuation mechanism is favoured by the exhibition of no or mild symptoms of disease in these reservoir hosts, which become infected with low-pathogenicity (LP) viral strains, mainly through the fecal-oral transmission route [2]. Highly pathogenic (HP) avian influenza viruses (AIVs) of subtypes H5 and H7 periodically emerge in domestic birds, in which LPAIVs of possible wild bird origin can shift to highly virulent strains [3]. The occurrence of outbreaks of disease caused by HPAIVs was considered a sporadic event in wild birds [4] until the emergence in 1997 of the HPAIV H5N1 strain, having pandemic potential for humans. After initial control measures, related to the effective mass slaughter of birds across the Hong Kong SAR, the HPAI H5N1 virus reemerged in Asian poultry flocks, and between late 2003 and early 2004 its progressive spread started among domestic and wild birds throughout areas of Eastern and South-East Asia, later involving the Asian portion of Russia, Europe, the Middle East, and Africa [5]. Morbility and mortality associated with the HPAI H5N1 infection in wild birds have provided a new scenario for evaluating the potential involvement of wild migratory avifauna in spreading and maintaining HPAIVs in natural habitats [6].

The Novosibirsk Region is important in influenza A virus ecology, as a part of the Asian portion of Russia representing the possible global epicentre of AIV persistence [7]. Huge numbers of wild birds, belonging to species considered a permanent reservoir of AIVs, migrate and congregate in breeding and/or moulting areas located in these Western Siberia wetlands. Major wild bird migration routes overlap in the Novosibirsk Region, mainly connecting this geographic area to the wintering territories of Eurasia, and Africa, as well as to more northern breeding sites in boreal coniferous forests and Arctic prairie [8]. In particular, the Central Asian Flyway covers large intracontinental territories of Eurasia and includes bird movements connecting Siberian lakes with Qinghai Lake (China) where in late April-early May of 2005, a new HPAIV H5N1 strain led to an anomalously high cumulative mortality of more than 6,000 wild birds [7]. The first reported HPAI H5N1 epizootics in the Russian Federation occurred at the end of July 2005 in backyard poultry flocks in the Novosibirsk Region, and a total of sixty-four and three H5N1 outbreaks occurred in this area in 2005 and 2006, respectively, when both wild and domestic bird species were affected [9]–[14].

A number of avian influenza virus (AIV) surveillance studies has been carried out in this Asian portion of Russia, during a span of time (2003–2011) including both pre- and post-H5N1 HPAIV epizootic periods [7], [15]–[16]. In this context, as shown in Table 1, different antigenic subtypes of AIVs have been isolated from wildlife and domestic hosts.

**Table 1:** Influenza A virus antigenic subtypes isolated from wild and domestic animal species in the Novosibirsk Region (2003–2011 period).

Antigenic subtype	VP	No. wild birds VI pos	No. wild mammals VI pos	No. domestic birds VI pos	Total No. VI pos	Years	References
H1N?	LP			12	12	2005	[16]
H1N1	LP	1			1	2011	[33]
H1N2	LP	1			1	2008	[8]
H2N2	LP	2	1		3	2003 *	[15] (VECTOR Institute, UD)
	LP	2			2	2004	(VECTOR Institute, UD)
H3N?	LP	3			3	2003	[15]
	LP			12	12	2005	[16]
H3N1	LP	1			1	2011	[33]
H3N2	LP	1			1	2011	[33]
H3N6	LP	1			1	2011	[33]
H3N8	LP	8			8	2008	[8]
	LP	8			8	2011	[33]
H4N?	LP	**1**		11	12	2005	[32], [16]
	LP	3			3	2011	[33]
H4N6	LP	3			3	2011	[33]
H5N?	LP	1			1	2003	[15]
H5N1	HP	**5**		**4**	**9**	2005	[10]–[12]
	HP	**1**			**1**	2006	[13], [18]
	LP	3			3	2011	[33]
H5N3	LP	1			1	2011	[33]
H6N2	LP	1			1	2011	[33]
H8N4	LP	2			2	2011	[33]
H13N2	LP	3			3	2011	[33]
H15N4	LP	1			1	2008	[7], [36]
H16N3	LP	3			3	2011	[33]
H?N1	LP	2			2	2011	[33]
H?N8	LP	1			1	2011	[33]
unsubtyped	LP	2			2	2008	[8]

VP, virus pathogenicity.

VI pos, virus isolation positive.

LP, low pathogenicity; HP, high pathogenicity.

* Underlining indicates year in which a specific HN subtype was prevalent, i.e. represented at least 25% of all influenza isolates for that year.

UD, unpublished data.

**In bold**, isolation data from a period in which H5N1 HPAI epizootics occurred.

**Figure 1:**
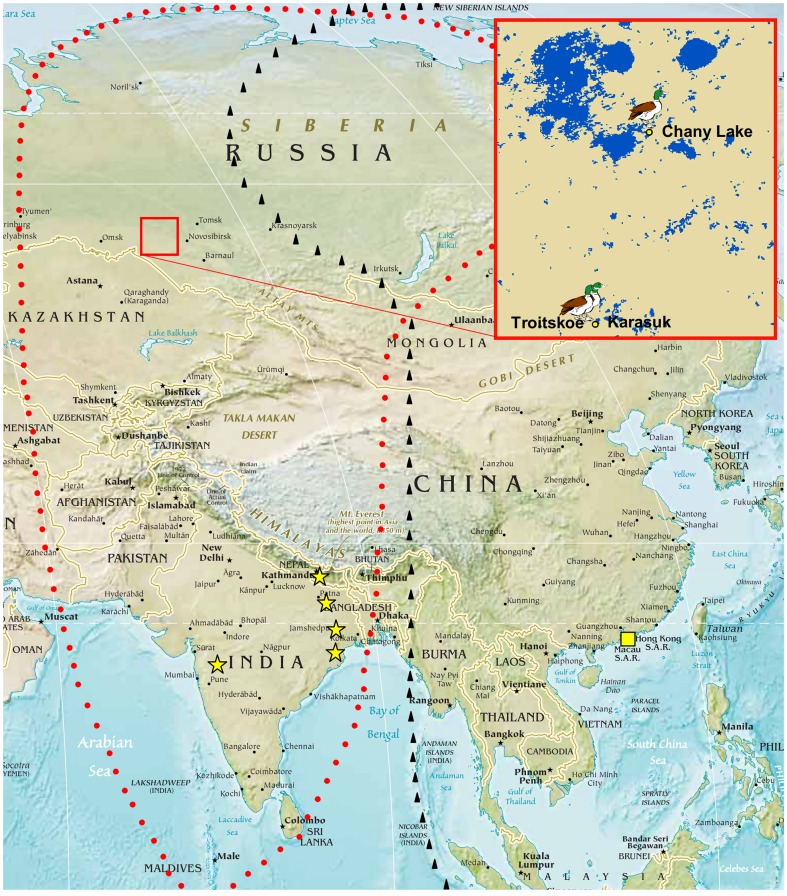
Bird sampling location and HP H5N1 Asian hotspots involving wild birds in 2012. During avian influenza virus surveillance activities, carried out in August-September 2012 in the Novosibirsk Region (South of Western Siberia, Russia), both cloacal and feather swabs were taken from each of the 166 wild and 152 domestic birds, sampled in the three localities of Chany Lake (54°36′58.79"N; 78°13′11.46"E), Karasuk (53°43′47.35"N; 77°52′02.70"E), and Troitskoe (53°43′47.45"N; 77°52′01.74"E). The richness of waterbodies and wetlands in the study area (blue-colored in the red box in Figure 1) provides ideal staging and moulting sites used by huge numbers of species of wild migratory aquatic birds. Areas bordered by red dots and black triangles include respectively the Central Asia Flyway (CAF) and part of the East-Asia Australian Flyway (EAAF), two major bird migration routes [23] connecting Siberian wetlands with geographic regions in which HP H5N1 outbreaks were detected in wild birds in 2012 [18]. The yellow stars within the CAF indicate HP H5N1 events in wildfowl, reported in different states of India and in Nepal (February 2012). The yellow square, within the EAAF, indicates eighteen HP H5N1 outbreak events occurring in China Hong Kong SAR in wild birds (January-July 2012). Information and image on Central Asian Russia (large map) were obtained on April 17, 2014 (as a part of “Physical Map of the World, August 2013”) from site https://www.cia.gov/library/publications/the-world-factbook/docs/refmaps.html maintained by the Central Intelligence Agency, Washington D.C. Source of image data product. Information and image on study area (small map) were obtained on February 3, 2014 from site https://lpdaac.usgs.gov/data_access maintained by the NASA Land Processes Distributed Active Archive Center (LP DAAC), USGS/Earth Resources Observation and Science (EROS) Center, Sioux Falls, South Dakota. (2012). Source of image data product.

In the present study, carried out in the summer of 2012 in the wetland ecosystems of the Novosibirsk Region, we evaluated the dynamics of circulation and/or introduction of AIV strains in avian populations of wild bird species ecologically linked to aquatic biotopes as well as in captive-reared waterfowl species representing a potential domestic reservoir of AIVs. Recent extensive surveillance studies suggested that wild migratory birds, showing ability to perpetuate LPAIVs in nature, are not competent as indefinite reservoirs of HPAIVs, carried by these infected hosts over small or moderate distances [17]. However, molecular evidences indicated that the HP H5N1 virus outbreaks occurred, in 2005 and 2006, in this Western Siberia area could have emerged from wild migratory birds [12]. In this context it is also notable that the HP H5N1 virus was isolated, in February 2012, from wild birds in India and Nepal [18], countries connected to the Novosibirsk Region by the Central Asian Flyway (Figure 1) [7]. Moreover, severe climatic conditions, occurring in this geographic area of Russia, could favour the preservation of AIVs in water and soils for long time periods [7], [19]–[20].

The present study on influenza A virus ecology was aimed to: i) improve the knowledge of AIV dynamics in this area, connected through bird migration routes to several HP H5N1 Asian hotspots; ii) investigate the potential AIV exchange between domestic and wild birds in the study area; iii) increase AIV detection ability by a novel sampling method. To do that, we examined avian species in natural and anthropic ecosystems, representing a possible wildlife/domestic interface. In particular, we used a new and more sensitive approach to AIV surveillance, obtained by integrating the conventional cloacal swab collection, aimed to show the fecal virus shedding, with the harvesting of samples from birds' plumage, aimed to detect viral particles concentrated onto birds' bodies [21]–[22].

Through an innovative approach to AIV surveillance, we showed lack of evidence for the environmental circulation of H5N1 HPAIV in the study area. We also confirmed the importance of Western Siberia wetlands in LPAIV ecology, providing evidence of changes in circulation dynamics of HN antigenic subtypes harboured in wild bird reservoirs.

## Materials and Methods

### Ethics statement

No wild birds were expressly killed or captured alive for this study. No specific permission was required for sample collection from shot wild birds, killed by local hunters in compliance with the Russian Federation hunting laws. Wild birds were captured alive during ringing activities conducted in the Biostation of the Institute of Systematics and Ecology of Animals, Siberian Branch of RAS (Chany Lake, Novosibirsk Region). No permit and approval were needed for sampling from wild birds captured alive and captive-reared waterfowl, because the study was conducted as part of the national avian influenza surveillance program. No endangered species included in the IUCN Red List of Threatened Species (available at: http://www.iucnredlist.org/) were involved in the field studies. In this study, no specific permissions were required to access to the Chany Lake, Karasuk, Troitskoe study area locations.

All animals were handled in accordance with the “Guidelines to the Use of Wild Birds in Research” (J.M. Fair, E. Paul, and J. Jones, Eds. 2010. Guidelines to the Use of Wild Birds in Research. Washington, D.C.: Ornithological Council, available at: www.nmnh.si.edu/BIRDNET/guide) drafted with reference to the guidelines issued by the Institutional Animal Care and Use Committee (IACUC).

The activities carried out using specimens were approved by the Committee on Biomedical Ethics, Research Center of Clinical and Experimental Medicine, Siberian Division of the Russian Academy of Medical Sciences, Novosibirsk (Protocol #25 from 19.11.2012).

### Study area and sample collection

Biological samples from three hundred eighteen birds were collected from August to September 2012 in three localities (Chany Lake: 54°36′58.79"N; 78°13′11.46"E; Karasuk: 53°43′47.35"N; 77°52′02.70"E; Troitskoe: 53°43′47.45"N; 77°52′01.74"E) of the Novosibirsk Region of Western Siberia. Richness of wetlands within the study area (Figure 1) provides an ideal habitat for wild aquatic birds and supports large breeding and migrating waterbird populations. In particular, the Lake Chany lacustrine system, characteristic of the Western Siberian forest-steppe, represents a key moulting site for huge numbers of post-breeding wild ducks, attracted here from a very wide geographical area [23]. Backyard waterfowl flocks for self-consumption and small farms of waterbirds, housed partially or totally outdoors, were also widespread in the study area, thus representing a sentinel model for the detection of AIV environmental persistence at the wildlife/domestic interface.

It is very important to note that the period under study matches the post-reproductive stage of the annual cycle of *Anseriformes*, that is characterized by a seasonal peak in AIV activity in these potential reservoir hosts. From an epidemiological point of view, this situation is related to the presence of large numbers of immunologically näive young birds, that are highly susceptible to avian influenza [1].

Overall, 166 wild birds of species ecologically linked to aquatic environment biotopes and belonging to the orders of *Anseriformes* (n. 118), *Charadriiformes* (n. 15), *Gruiformes* (n. 24) *Passeriformes* (n. 4), *Podicipediformes* (n. 5), together with 152 captive-reared waterbirds (n. 105 domestic geese, n. 45 ducks, n. 2 swans) were sampled. Wild birds were captured alive by mist nets or shot in the course of hunting, whereas all the domestic birds, reared in small commercial farms representing a major part of the family's income, were sampled while alive. Wherever possible, birds under examination were aged and sexed according to Baker [24] and Svensson [25] and then categorized into juveniles (individuals hatched during the last breeding season) and adults (individuals hatched any year before the last breeding season). Bird species analyzed, as well as their age class distribution are shown in Table 2.

**Table 2:** Analyzed bird species and age class distribution (Novosibirsk Region, August-September 2012).

Order	Species	Status	No.	Age		
			individuals	HY	AHY	UA
*Anseriformes*	*Anas platyrhynchos dom.*	C-R	9	6	1	2
	*Anas platyrhynchos*	W	16	16		
	*Anas platyrhynchos*	C-R	34	32	2	
	*A. platyrhynchos x A. platyrhynchos dom.*	C-R	2	1		1
	*Anser anser dom.*	C-R	105	102	3	
	*Cygnus cygnus*	W	7			7
	*Cygnus olor*	C-R	2		2	
	*Aythya ferina*	W	13	9	1	3
	*Anas strepera*	W	19	9	1	9
	*Aythya fuligula*	W	3	2		1
	*Anas crecca*	W	48	14	5	29
	*Anas querquedula*	W	2	1		1
	*Anas acuta*	W	2	2		
	*Anas clypeata*	W	8	4		4
*Gruiformes*	*Fulica atra*	W	23	10	12	1
	*Porzana pusilla*	W	1			1
*Charadriiformes*	*Gallinago gallinago*	W	1			1
	*Charadrius hiaticula*	W	1			1
	*Lymnocryptes minimus*	W	1			1
	*Xenus cinereus*	W	1			1
	*Philomachus pugnax*	W	6			6
	*Tringa glareola*	W	3	1		2
	*Larus ridibundus*	W	2			2
*Passeriformes*	*Corvus frugilegus*	W	1			1
	*Motacilla flava*	W	1			1
	*Acrocephalus agricola*	W	2			2
*Podicipediformes*	*Podiceps nigricollis*	W	1	1		
	*Podiceps cristatus*	W	4		2	2
Total			318	210	29	79

HY, hatching year; AHY, after hatching year; UA, undetermined age.

C-R, captive reared; W, wild.

dom., domesticus.

To increase the sensitivity of detection of AIVs circulating in natural and anthropic environments, we used a novel approach to AIV surveillance, obtained by integrating the conventional cloacal swab collection with the harvesting of samples from birds' plumage [21]–[22]. This latter sampling method, based on the collection of swabs rubbed over feathers located on the bird's breast and flanks [21], allows the detection of AIVs concentrated from aquatic environment to bird bodies. Whereas analyses of cloacal swabs reveal viruses shed by infected ducks, viruses isolated from feathers may not have infected the sampled individual yet and just come from the environment. As previously reported by Delogu et al. [21] and Lebarbenchon et al. [22], virological examination of feather samples can reveal the presence of birds testing “false-negative” by conventional AIV surveillance system (virus isolation negative from cloaca) but carrying infectious virus on their plumage. Both cloacal and feather samples were collected from each bird and separately placed in 2 ml individual tubes with transport medium containing phosphate buffered saline:glycerol (1∶1) with antibiotics, prepared as previously described [26]. Immediately after collection, virological samples were stored at −196°C in liquid nitrogen until laboratory testing.

### Virus isolation and characterization

Cloacal and feather swabs were tested for influenza viruses by virus isolation in embryonating specific-pathogen-free (SPF) chicken eggs, according to standard procedures [27]. Inoculated allantoic fluid was examined using the haemagglutination (HA) assay performed with 0.5% chicken red blood cells as previously described [27]. Isolates were identified by the serologic haemagglutination inhibition (HI) assay [27] and by polymerase chain reaction (PCR) with the use of primers for haemagglutinin and neuraminidase subtyping [28]–[29].

### Analysis of data

#### Virus subtype prevalence

To better quantify the temporal distribution of AIVs circulating in the study area, we identified the most representative antigenic subtypes isolated in the present study (Table 3) as well as those previously detected (Table 1). In each year, we considered as prevalent the most frequently detected HN subtypes, representing at least 25% of all influenza isolates for that year.

**Table 3:** Viruses isolated from cloacal and/or feather samples collected from wild ducks (Western Siberia, August-September 2012).

Bird No.	Species	Sex	Age	VI positive samples	Virus	Subtype
1	*Anas crecca*	F	Ad	CS	A/eurasian teal/Siberia/17/2012	H4N?
2	*Anas crecca*	M	Juv	CS	A/eurasian teal/Siberia/21/12	H3N8
3	*Anas acuta*	F	Juv	CS	A/pintail/Siberia/37/2012	H3N8
4	*Anas platyrhynchos*	F	Juv	CS	A/mallard/Siberia/75/2012	H4N6
5	*Aythya ferina*	F	Juv	FS	A/pochard/Siberia/97/2012	H4N6
6	*Anas clypeata*	M	Juv	FS	A/shoveler/Siberia/99/2012	H4N6
7	*Anas clypeata*	F	Juv	FS	A/shoveler/Siberia/101/2012	H4N6
8	*Anas crecca*	F	Juv	CS	A/eurasian teal/Siberia/104/2012	H4N6
				FS	A/eurasian teal/Siberia/105/2012	H4N6
9	*Anas crecca*	F	Juv	CS	A/eurasian teal/Siberia/106/12	H4N6
				FS	A/eurasian teal/Siberia/107/2012	H4N6
10	*Anas crecca*	U	U	CS	A/eurasian teal/Novosibirsk/9c/2012	H4N?
11	*Anas crecca*	U	U	FS	A/eurasian teal/Chany/714/2012	H3N8
12	*Anas crecca*	U	U	FS	A/eurasian teal/Novosibirsk/776/2012	H?N2
13	*Anas platyrhynchos*	U	U	FS	A/mallard/Novosibirsk/794/2012	H2N?
14	*Anas platyrhynchos*	U	U	FS	A/mallard/Novosibirsk/407/2012	A+
15	*Anas platyrhynchos*	U	U	FS	A/mallard/Novosibirsk/408/2012	A+

VI, virus isolation.

Juv, juvenile birds (hatching year age); Ad, adult birds (after hatching year age); U, undetermined.

CS, cloacal swab; FS, feather swab.

#### Statistical analysis

The Fisher's exact test was used to test, within each taxonomic order, differences in proportion of wild or captive-reared birds shedding AIVs from cloaca, and determine the statistical significance (p<0.05) of differences in results by age, and sex. Because of the relatively small sample of aged and sexed birds, the Fisher's exact test was chosen for contingency tables analysis with small expected cell frequencies. Data were analyzed using R language (http://www.r-project.org/).

## Results

### Virus isolation and characterization

To better investigate the environmental circulation of AIVs in natural and anthropic ecosystems of Western Siberia, both cloacal and feather samples were collected from each of the 318 birds examined (Table 2). Overall, 17 influenza A viruses were identified in biological samples, tested by virus isolation assay in embryonating eggs. As shown in Table 3, virus isolates were obtained from wild *Anseriformes* captured, over the period August-September 2012, in the Novosibirsk Region. The prevalence of virus isolation ranged from 10.2% (17/166), when calculated on the overall sample of wild birds, to 14.4% (17/118), when calculated on wild *Anseriformes* species, only. A total of 15 wild ducks tested virus isolation (VI)-positive and, in detail, 5 ducks were VI-positive from cloaca only (*Anas crecca*, n. 3; *Anas acuta*, n. 1; *Anas platyrhynchos*, n. 1), 8 ducks from feathers only (*Aythya ferina*, n. 1; *Anas clypeata*, n. 2; *Anas crecca,* n. 2; *Anas platyrhynchos*, n. 3), and 2 ducks (*Anas crecca*) were VI-positive from both feathers and cloaca. The proportions of wild *Anseriformes* tested VI-positive from cloaca and feathers were 5.9% (7/118) and 8.5% (10/118), respectively. The overall prevalence of virus isolation, obtained from both sampling methods, was 2.4 times higher than that calculated on results from cloacal swab examination only (14.4% *vs* 5.9%, respectively).

According to the serological and molecular characterization, AIV isolates included three H3N8, eight H4N6, two H4N?, one H2N?, one H?N2, and two unsubtyped LP viruses. The inability to characterize some isolates (Table 3) is likely due to the detection of emerging AIVs, whose mutations could not allow the subtyping by using available reagents.

Both cloacal and feather samples collected from 152 captive-reared *Anseriformes* (Table 2) were tested negative by virus isolation assay.

No H5N1 HPAIV was isolated during the study period. According to sample sizes of wild and domestic birds (166 and 152, respectively) the presence of the infection up to the lowest prevalence level of 2% could be ruled out in the examined populations, when using 95% confidence level [30].

### Analysis of data

The H4N6 antigenic subtype, detected in four out of five VI-positive duck species, was found to be the prevalent one, representing 47.1% of all influenza A isolates for the 2012 study period (Table 3). By analyzing virological data obtained in the study area between 2003 and 2011, the H2N2 antigenic subtype resulted to be prevalent in 2003 in wildlife species, including muskrat (*Ondatra zibethicus*), whereas the H3N8 antigenic subtype was found to be prevalent in wild birds in 2008 and 2011 (see underlined years in Table 1).

No age- and sex-related differences in proportions of wild *Anseriformes* tested virus isolation positive from cloaca (Table 2 and Table 3) have been detected.

## Discussion

As previously reported (see Table 1 for references), during the period 2003–2011, one hundred and one influenza A viruses were isolated in the Novosibirsk Region from wild birds (n. 61 isolates), wild mammals (n. 1 isolate), and domestic birds (n. 39 isolates). The detected virus strains belonged to H1 (n. 14), H2 (n. 5), H3 (n. 34), H4 (n. 18), H5 (n. 15, including both LP and HP viruses), H6 (n. 1), H8 (n. 2), H13 (n. 3), H15 (n. 1), H16 (n. 3), and uncharacterized H (n. 5) subtypes, showing a variable prevalence of circulation in wildlife hosts (in which all antigenic subtypes were detected) and domestic birds (harbouring H1, H3, H4, H5 antigenic subtypes, only). In particular, among influenza A viruses identified by serologic and/or genetic characterization of both haemagglutinin and neurminidase proteins, the H2N2 antigenic subtype resulted to be prevalent in 2003 in wildlife species, including muskrat (*Ondatra zibethicus*), whereas the H3N8 antigenic subtype was found to be prevalent in wild birds in 2008 and 2011 (see underlined years in Table 1).

In the course of the present research, conducted in 2012, we evaluated AIV persistence in natural and anthropic ecosystems of the Novosibirsk Region, by examining both cloacal and feather samples collected from wild birds and domestic waterfowl. No HPAI H5N1 virus was found in this study area, connected through bird migration routes to epidemiologically relevant H5N1 hotspots taking place in Asia [18] (Figure 1), whereas 17 LPAIVs were detected in wild *Anseriformes*. Whereas comparable proportions of wild *Anseriformes* tested virus isolation positive from cloaca and feathers (5.9% *vs* 8.5%, respectively) were detected, the overall prevalence of virus isolation was 2.4 times higher than that obtained from cloacal swab examination only (14.4% *vs* 5.9%, respectively).

As previously reported [21]–[22], our data emphasise the usefulness of the combined cloacal/feather sampling approach, able to improve the virus isolation sensitivity by allowing the detection of individuals with active virus replication and shedding, as well as birds testing “false-negative” by conventional AIV surveillance system, because carrying infectious virus on their plumage only. Thus, during the time period between the virus adhesion to the bird's body and the infection (possibly due to self- and/or allopreening) AIVs, including the highly pathogenic ones, could move in nature by a circulation mechanism in which the epidemiologic status of “uninfected birds carrying AIVs on their feathers” certainly does not affect the fitness of the host [21].

To our knowledge, the present study is unique in documenting by this new sampling approach, the environmental circulation of AIVs in a study area considered at risk for the HP H5N1 virus emergence [11], persistence [20] and/or re-emergence from migratory birds [7], [31]. Taking this into account, our findings agree with previous studies showing a lack of circulation of the HP H5N1 virus in wild birds of the Novosibirsk Region, after the last outbreak in 2006 [7], [32]–[33]. In particular, even if a virus circulation occurring at low prevalence level (<2%) can not be ruled out (See Results section), a number of conditions, such as i) the combined (cloacal/feather) sample collection methods, ii) the study period characterized by the presence of numerous juvenile waterbirds (immunologically näive and highly susceptible to AIVs), iii) the concurrent absence of reports of mortality cases in wild or domestic birds (possibly related to HPAIV outbreaks), provide further evidence for the H5N1 possible extinction and lacking reemergence in the study area. Despite environmental and climatic conditions could favour the virus survival in Western Siberia wetlands [7], [19]–[20], viral survival is highly dependent on host fitness. From an evolutionary ecology point of view, HPAIV infections in wild bird populations tend to be self-limiting, unlike those caused by LPAIVs, perpetuated in avian reservoirs in a well-adapted host/parasite balance [3], [17]. The H5N1 virus extinction in natural habitats is further supported by negative results for virus isolation in captive-reared waterfowl, consisting of more than 90% juvenile birds housed in environmental conditions which could favour host-pathogen interaction at the wildlife/domestic interface. These birds, mainly raised by a family or in a household, were reared in rural areas near wetlands, in small scale farms in which they were housed partially or totally outdoors, under relatively poor bio-security conditions. In this context, passerine birds such as sparrows or magpies, could represent a potential ecological bridge between the captive-reared birds and wild bird reservoirs of AIVs, possibly attracted by the presence of ponds and canals in areas surrounding the farms. In addition to potentially infected birds, fomites moved by humans could allow the introduction of AIV contaminate feces.

As previously showed (Table 3), seventeen LPAIVs were isolated during the present study from wild *Anseriformes*, including three H3N8, eight H4N6, two H4N?, one H2N?, one H?N2, and two unsubtyped viruses. In particular, the H2 antigenic subtype was found again in wildlife populations, after the last detection in 2004 (Table 1). Moreover, the H4N6 antigenic subtype, previously detected at low prevalence level in wild avian species (Table 1), was found to be the prevalent strain isolated in 2012. These data, seen against the background of previous AIV surveillance studies carried out since 2003 (Table 1), provide evidence of a temporal variation in the prevalence of AIV antigenic subtypes circulating in this study area. As a matter of fact, the H3N8 higher prevalence level recently detected in wild birds (Table 1), seems to be replaced by that of the H4N6 antigenic subtype, representing 47.1% of all influenza A viruses isolated from wild birds during the summer 2012. Our findings agree with previous multi-year studies [34]–[38], showing a cyclic nature of AIV subtype circulation in waterfowl populations. In this context, the lack of age-related difference in proportions of wild *Anseriformes* tested virus isolation positive from cloaca, could be explained by the absence of a specific herd immunity in both adult and juvenile birds, possibly related to the recent emergence, in wild populations, of the H4N6 antigenic subtype. The main limitation of this paper is the lack of in-depth characterization of viruses, isolated from birds' body surface and cloaca (Table 3). Preliminary results, based on partial sequences of HA and NA genes, showed a high level of similarities between H4N6 strains isolated from feather and cloacal swabs taken from single individuals (data not shown). More detailed studies will be useful to provide further valuable information of the antigenic and nucleotide sequence similarities between AIVs circulating in the study area.

In contrast to what observed in wild *Anseriformes*, both cloacal and feather samples collected from domestic birds (Table 2) tested virus isolation negative. The absence of virus detection in captive-reared waterfowl, potential reservoir species of LPAIVs, could be explained by basic biosecurity measures adopted, together with the still ongoing vaccination policy, as a consequence of the 2005–2006 HP H5N1 outbreaks [12]. In that context, farmers adopted the use of pens to mitigate contacts between domestic and wild birds, as well as they prevented domestic waterfowl grazing in wetlands surrounding the farms.

Our findings emphasise the importance of Western Siberia wetlands in influenza A virus ecology and epidemiology, and provide evidence of changes in circulation dynamics of AIV antigenic subtypes harboured in wild bird reservoir populations. In this context we did not find any HP H5N1 virus, explainable by the poor environmental fitness of HPAIVs in natural ecosystems. Changes in virus circulation dynamics, strictly related to bird migrations as well as to the intrinsic mutation ability of influenza A viruses, can lead to the emergence of novel and/or rare AIV strains, such as the reassortant influenza H15N4 virus subtype [8], [39], previously detected in this area (Table 1). All the above circumstances fit in the influenza A virus evolution and adaptation to novel hosts, and further antigenic and molecular characterization of isolates from animal species could provide additional information to better understand the Siberian fauna's role in the global ecology of influenza A virus. In particular, in-depth studies of isolates, based on bioinformatic approaches to virus molecular evolution and phylogenesis, will be needed to better elucidate mechanisms involved in AIV perpetuation in this area.
